# Nurse anesthetists’ perceptions of heat conservation measures in connection with surgery – a phenomenographic study

**DOI:** 10.1186/s12912-023-01508-1

**Published:** 2023-09-18

**Authors:** Ingrid L. Gustafsson, Carina Elmqvist, Bengt Fridlund, Kristina Schildmeijer, Mikael Rask

**Affiliations:** 1 Faculty of Health and Life Sciences, Linnaeus University, Växjö, S-351 95 Sweden; 2 Centre of Interprofessional Collaboration within Emergency Care (CICE), Linnaeus University, Växjö, S-351 95 Sweden; 3 Faculty of Caring Science, Work Life and Social Welfare, University of Borås, Borås, S-501 90 Sweden; 4 Department of Research and Development, Region Kronoberg, Växjö, 352 57 Sweden; 5 Faculty of Health and Life Sciences, Linnaeus University, Kalmar, S-391 82 Sweden

**Keywords:** Experience, Hypothermia, Perception, Perioperative nursing, Phenomenography, Warming

## Abstract

**Background:**

To minimize the risk of perioperative hypothermia, it is recommended that healthcare professionals be familiar with heat conservation measures and use passive and active warming methods, in line with international guidelines. However, there is a low level of adherence perioperatively to the use of heat conservation measures. To understand why, there is a need to capture the nurse anesthetists’ perspective. The aim is to describe nurse anesthetists’ perceptions of heat conservation measures in connection with surgery.

**Methods:**

An inductive descriptive design with a phenomenographic approach was chosen. A total of 19 nurse anesthetists participated and were interviewed. Data were analyzed according to Larsson and Holmström’s phenomenographic seven-step model.

**Results:**

Six ways of understanding the phenomenon heat conservation measures in connection with surgery were found: the preventive, the useable, the untenable, the caring, the adaptive, and the routine care approach. These approaches were related to each other in a flexible way, allowing for several to co-exist at the same time, depending on the situation.

**Conclusions:**

Nurse anesthetists want to prevent the patients’ heat loss and maintain normothermia, regardless of the type of surgery. This willingness, motivation, and intention enable the use of heat conservation measures. However, there are perceptions that have an impact, such as doubts and uncertainty, access, time and financial constraints, preconditions, routines or habits, and lack of availability of education/training. These barriers will require support from an organizational level to promote lifelong education and guidelines. As well as offer education at the nurse anesthetists’ program.

## Background

It is common for patients to develop perioperative hypothermia during the perioperative period, i.e., a body temperature below 36º C [1–3], and this increases the risk for several adverse events [4]. From the patients’ perspective, cold and chills are the most difficult experiences in operating theatres [5]. Furthermore, the feelings of temperature comfort are individual, and when needs are met, a sense of well-being, calmness, and security occurs [6]. To minimize the risks of perioperative hypothermia, maintain normothermia, and ensure the thermal comfort of the patient, healthcare professionals are recommended to use passive and active warming methods in current guidelines [7–9], i.e., heat conservation measures.

Passive warming, also known as thermal insulation, means isolating the patient’s body from cold and preventing heat loss by minimizing exposure, which can be done with shirts, caps, socks, leg covers (a quilt shaped like a leg sack), and blankets or quilts [7–10], preferably warmed in a cabinet. Furthermore, an operating room temperature of 21º C also provides passive warming [8, 9, 11]. Active warming means giving the patient an active warming prevention such as warm intravenous or irrigation fluids, which reduce the risk of patients’ heat loss [7–9, 11, 12]. A relatively new active prevention is the self-warming blankets with air-activated warming pads, providing active warming for anywhere from 30 min to 10 h [13]. Furthermore, resistive/conductive heating such as a warming blanket/mattress coupled to a heating unit can be used [14], however, there is a lack of knowledge if the mattresses can impact patients’ risk for pressure ulcers. The most effective and cost-effective prevention is forced-air warming [8, 9] which provides active warming by heated air. However, the most effective method to avoid heat loss is to pre-warming patients before anesthesia induction, due to the smaller temperature gradient between peripheral and core temperature, which minimizes temperature drops [7–9, 11, 14].

Despite there being evidence-based guidelines, studies show a low level of adherence to the use of some heat conservation measures [1, 15, 16]. Preoperatively 5% of patients received a prewarmed operating table [16], 61–73% of patients received warming blankets [15, 16], and 5–20% forced-air warming [1, 15]. Intraoperatively 34–64% of patients received forced-air warming [1, 15–17] and 9–67% received fluid warming intraoperatively [1, 16]. Furthermore, 31% of patients received padded leg covers [16]. Postoperatively 9% received forced-air warming [15].

The responsibility to provide heat conservation measures, measure patient body temperature, and maintain normothermia is included in anesthesia care [18, 19]. Whether the responsibility is the nurse anesthetist, the anesthesiologist, or the anesthesia nurse, depends on the country in question, due to nurses’ varying competence in the field. In Sweden, a nurse anesthetist is a registered nurse with a postgraduate degree in specialist nursing anesthesia care and a one-year master’s degree. They have the competence as well as the legal authority to induce, maintain, and carry out regional and general anesthesia independently, or in collaboration with an anesthesiologist, depending upon the patients’ ASA score [20]. The nurse anesthetists provide anesthetic nursing that is person-centered and characterized by three attributes: *keep in touch with the patient, watch over the patient, and being one step ahead* [21].

According to the nurse anesthetists’ professional role, in terms of avoiding perioperative hypothermia, nurse anesthetists should monitor temperature and identify and prevent complications [20] by using temperature devices and heat conservation measures [7–9]. However, in the few existing studies [1, 15, 16] the rate of use of heat conservation measures was low, and questions arose as to why some patients did not receive heat conservation measures and the rationale. Among the reasons offered were that heat conservation measures were time-consuming, impractical to use, and that they affected surgeons’ access to the patient. Additional reasons were the lack of heat conservation measures, that such measures were not routine, that the surgery was short or that the operating wound was small [16]. Moreover, in Canada, anesthesiologists felt insecure with the guidelines’ content of perioperative temperature management and a lack of access to heat conservation measures influenced their ability to provide them. They also doubted whether their management was skilled enough [22]. In this area, there is a shortage of knowledge from the nurse anesthetists’ perspective to understanding what affects nurse anesthetists use of heat conservation measures - the relationship between the nurse anesthetists and heat conservation measures. To our knowledge, no previous study has been made of nurse anesthetists’ perceptions in this area. The answer can help future implementation and it is thereby a need to capture how nurse anesthetists perceive heat conservation measures. Therefore, this study aims to describe nurse anesthetists’ perceptions of heat conservation measures in connection with surgery.

## Methods

An inductive descriptive design with a phenomenographic approach was used [23] to describe variations of nurse anesthetists’ perceptions of the phenomenon: heat conservation measures in connection with surgery. Phenomenography was developed in order to understand a phenomenon from a qualitative and nondualist approach. This nondualist ontology means that humans and the world are internally related to each other, and an interrelationship between object and subject exists [24]. Perception is a human understanding that appears between the human and the world [25], i.e., in this study, the nurse anesthetists and the phenomenon, the heat conservation measures studied. Phenomenography describes common humans’ different ways of understanding a phenomenon [24]. In phenomenography, there are two perspectives of the phenomenon: the first-order perspective is a statement about what things are [23, 26] referred to as the referential aspect (what) [24], and the second-order perspective is a statement about what things appear to be [23, 26] referred to as the structural aspect (how) [24].

### Settings

Based on the relative size of Swedish hospitals, four hospitals were chosen, one district, two regional, and one university hospital, with 6–33 operating theatres respectively. They collectively represented all geographic areas of Sweden, from east to west and north to south, to reflect the national context. The heat conservation measures used in Sweden are as follows: cotton gowns, caps, leg covers, cotton blankets or disposable blankets, quilts, resistive mattresses, and forced-air warming into gowns, mattresses, or blankets, as well as active heated intravenous and irrigation fluids.

### Participants

At each hospital, the directors for the operation departments’ organization gave written approval and provided contact persons. This person gave the first author access to give information verbally at workplace meetings for nurse anesthetists in person, or by Skype and afterwards forwarded an e-mail with written information. The contacts also announced reminders via e-mail or in person. The inclusion criteria were nurse anesthetists with more than one year of experience in an operating department who were thereby familiar with the phenomenon. The strategic sample was comprised of a total of 19 nurse anesthetists, 14 women, and five men, aged 28–66 years, with 1–41 years of experience as a nurse anesthetist (Table 1).

**Table 1:** Socio-demographic and work experiences characteristics of the nurse anesthetists (n = 19)

Characteristics	Categories	n	
Gender	Female	14	
	Male	5	
Age	28–30	1	
	31–40	6	
	41–50	4	
	51–60	4	
	61–66	4	
Work experiences as nurse anesthetist	1–5	6	
	6–10	2	
	11–15	3	
	16–20	1	
	21–25	3	
	26–30	1	
	31–35	1	
	36–41	2	

### Data collection

The interview guide started with socio-demographic data regarding age and professional experience. This was followed by a question regarding what heat conservation measures they were familiar with. Then three main questions were asked: What do heat conservation measures mean to you? What are your experiences with patient’s needs for heat conservation measures? How do you use heat conservation measures? Follow-up questions were also asked, such as, *Could you explain in further detail? You said …. what do you think about that?* A pilot interview was conducted with a nurse anesthetist, to test the semi-structured interview guide. The pilot interview was not included, due to no employment at any of the participating hospitals. However, it revealed some uncertainties regarding the terms passive and active warming, so these terms were changed in the interview guide to the concrete and generic terms in a nurse anesthetist’s daily work, heat conservation measures. All participants were interviewed by the first author in a separate room at their workplace. The recorded interviews lasting 16–55 min (average 29) were transcribed verbatim and anonymized for all the other authors by the first author. Data were collected from June 2018 - February 2019.

### Data analysis

The analysis was performed by the first author according to Larsson and Holmström’s phenomenographic seven-step model [27], Table 2. The co-authors, having extensive experience within this phenomenographic approach, followed the systematic process, and reflected on and verified the outcome of each step in the analysis. The result is presented in description categories with quotations marked with a number within parentheses for each participant.

**Table 2:** The data analysis process according to Larsson and Holmström’s phenomenographic seven-step model [27]

Steps	Process
Step 1	The first author read the whole text.
Step 2	During the next reading, she marked the nurse anesthetists’ answers to the three main questions in the text.
Step 3	These marks were then discussed with author MR and BF to find the dominant way of understanding, the focus, and how it was described. Then the first author wrote preliminary descriptions for each interview.
Step 4	These descriptions were then grouped together in categories based on differences and similarities by the first author, which were then reflected upon in the research team.
Step 5	Non-dominant ways of understanding were identified by the first author, and then discussed with author MR and BF.
Step 6	The first author reflected upon a structure in the outcome space, which was then reflected upon in the research team.
Step 7	Each of the six understandings based on the descriptions were assigned a metaphor by the first author in agreement with the research team.

### Ethical consideration

Reflections were done in the research team according to the Helsinki Declaration [28]. All participants gave their written consent after receiving information verbally and in writing about the study design, objective, and confidentiality as well as the possibility to withdraw their consent if necessary.

## Results

Six ways of understanding emerged: the preventive approach, the useable approach, the untenable approach, the caring approach, the adaptive approach, and the routine care approach (Table 3). These approaches were related to each other in a flexible way allowing for several to co-exist depending on the situation.

### The preventive approach

The preventive approach contained perceptions of being one step ahead, with the goal of protecting the patients from heat loss and maintaining normothermia regardless of surgery to minimize complications, discomfort, and mortality. This responsibility was perceived as a failure if not achieved.

*It is for the patient that we are here, for the patient, [is our focus] and it is my damned duty, as I say, to make sure that this is done well, and that I do it in the best way (12).*

In order to meet the goal, measuring the temperature was perceived as one tool for surgeries lasting longer than 30 min, to be able to make decisions concerning which and to what extent heat conservation measures should be used, or adjusted. However, there were perceptions of skepticism and frustration regarding the reliability of tympanic temperature devices and their error values. The consequence was that the clinical gaze was considered the most important for observation of the patient, together with the temperature.

*You feel at the patient’s peripheral. You feel their head, how does the face look, has it begun to bead or sweat? // You not only measure but rather feel the body and determine if it is sweat or dry or cold peripheral // so that is the tool, besides measuring temperatures (10).*

There were also perceptions that data other than temperature needed to be collected for decision support for heat conservation measures during the overall process, in the perioperative period, including age, ASA physical status classification, BMI, anesthesia, and estimated time.

*A surgery takes 30 min, but that is actual operating time // then the patient has to come into the operating theatre and be connected to the monitor// the operating theatre nurse has to scrub and drape, and that takes 60 min and then I go to wake up the patient (2).*

### The useable approach

The useable approach contained perceptions of doubt and uncertainty regarding for how long heat conservation measures maintain the warmth when taken from a cabinet, due to a lack of evidence of effectiveness. However, at the same time, there were perceptions that these heat conservation measures did not pose harm to the patient, and they had an isolating function and were thus used.

*It loses effect, then I think we have a long, half-meter tubing connected to the patient and it has to lose a lot of warmth from that // I have to think about it, it must be better than nothing (1).*

Furthermore, there were perceptions of uncertainty and a fear of burning patients, especially children, when using active self-warming blankets, due to split opinions regarding whether and how to use them. Uncertainty also existed regarding the preventive effect of using operating table mattresses with a resistive mattress on top, especially in hip surgeries, due to a high level of pressure on the hip and shoulder. Furthermore, perceptions of being hindered by lack of access and knowledge concerning heat conservation measures that could be facilitated by guidelines with a common goal.

*They [university] did not talk so much about heat conservation measures so what I have learned is or caught is what I have got from my workplace (8).*

There were, however, perceptions of overall appreciation regarding the effectiveness of resistive mattresses for all involved in the intraoperative process. The effectiveness of self-warming blankets when used for warming the patient’s bed before, during, and after the surgery was appreciated, as well as forced-air warming of the patient which, however, had a disturbing noise.

*One thing is that we are warming the beds before the patient ends up in its own bed, so again that is positive (10).*

Furthermore, there were contradictory perceptions of disposable blankets as both hygienic and fresh, however, a waste of environmental resources as well as ineffective due to their lightness and flimsiness, requiring them to be tucked around the patient to provide warmth.

*If you tucked them in, then trick is that you tucked it in under the patient around the legs and around the body, otherwise it not warms them (4).*

### The untenable approach

The untenable approach contained perceptions of hindrances, like working on an assembly line in brief surgeries, having a small amount of time to prepare for forced-air warming, having no time for warming up before awakening, and thus not being able to use heat conservation measures as intended.

*One thing is that it should go fast ….changes…it should go super-fast nowadays, you have people that clock you // if it is fast tempo, then you don’t think about it, you just go for it (5).*

There were also perceptions of not obtaining the right preconditions, such as a need for assistance and materials, together with financial constraints. A further perception was that it was difficult to maintain normothermia in an already chilled patient.

*The patient is often cold when they arrive to us before we even begin to cool them down here, and that is a problem when we already have to work uphill as you say (11).*

### The caring approach

The caring approach contained perceptions that before and after general anesthesia, both temperature comfort and body temperature were prioritized, while during the anesthesia was more focus given to the physiological body temperature. Moreover, it was important to remain observant of reactions of temperature (dis)comfort during the awakening phase. This responsibility lasted until the patient was fully conscious. Furthermore, the clinical gaze was important during the whole process in order to see and listen to the patient’s unspoken needs. There were perceptions that experience-based knowledge enabled reading the patient’s facial expression and body language.

*You have to look for other things if you can see anything in facial expressions or if they look at you when you speak with them or … high blood pressure or a fast pulse or if you can find any clue somewhere // the patient is the most important means to catching it (10).*

There were perceptions that offering information about experiencing warmth and coldness increased preparedness for the situation, and decreased suffering, creating feelings of security and comfort. Furthermore, tucking in the patient was perceived as more important than the warmth itself.

*It is more like a caring action than an attempt to warm the patient. this is just to create some well-being in the situation to beds down and tuck in (14).*

Moreover, warming was perceived to do much for the patient and cost little. Furthermore, it was perceived as good for the soul to be given warmth, and that being warm and cozy, as the last recollection before being anesthetized, had to be of importance.

*The warm blanket I think is really good for the soul …a human need, I think is really important (19).*

### The adaptive approach

The adaptive approach contained perceptions of avoiding disagreement among the team, in favor of a good atmosphere during the surgery.

*Even if I want to claim my part of the world and do it as well as possible, I still believe that we are a team that should work for the patient and that would be better if we could communicate without quarreling with each other, so can I be flexible and find solutions that please everybody, so I find out how to do that (12).*

There were perceptions of being split between the patients’ need for warmth, the team members’ expressions of risk for post-operative wound infections, and the manufacturers’ statement of no risk.

*The operating theatre nurse says that it swirls particles in the operation wound all around when they have scrubbed and stuffed well, we do not have any science behind that, so I have no idea (13).*

Adaption with other heat conservation measures was done due to perceptions of lacking evidence-based knowledge, which caused insecurity and a fear of complications affecting the patient. Despite experiences of delays in activating forced-air warming, which made the patient drop in temperature, this took hours to recover from.

Furthermore, there were perceptions of a split of professional responsibility between the patient, surgeon, and the temperature level in the operating theatre. The need to minimize heat loss for the patient conflicted with the interest of a comfortable work environment temperature for the surgeon. The nurse anesthetists thereby adapted and provided additional and varied heat conservation measures if the temperature level in the operating theatre was lowered. Accordingly, there was an uncertainty regarding whether the surgical team became too warm or the patient became too cold, affecting the patient’s safety.

*There is also one like this if you look at patient safety, what is really more dangerous, that it is 21 degrees in the operating theatre and that they feel comfortable while operating, than that it is 22 degrees in the operating theatre and the patient does not get as cold, but that they [surgeon] have a hard time focusing on the task because they are sweating; that is very difficult to know (14).*

### The routine care approach

The routine care approach contained perceptions that all patients received warm intravenous fluid and warm blankets from cabinets as a routine. Furthermore, there were perceptions that only older, frail patients, patients receiving certain types of surgery, and patients who were freezing were given more measures, such as caps, leg covers, and forced-air blankets.

*I’m trying to think about the patients as best I can, but I’m probably a little used to the fact that open abdomens are the ones that we warm up the most…. then we have our…. uh collum fracture patients or those here who are getting hip surgery, they got heat often… old fragile patients you usually have use heat on // I do not know why I do not have what is traditional, and there are old habits here you have always done and then you continue (5).*

Moreover, there were perceptions that the length of surgery, longer than 30–60 min required active heat conservation measures, such as forced-air, resistive mattresses and/or electric blood and fluid warmer. Short surgery time was seen as a less dangerous situation, with minor risks for heat loss and a decreased need for warmth. For others, there were perceptions of habitual carelessness, when not using heat conservation measures in short surgeries. Even if the information about heat conservation measures was up to date, it was easy to fall back into old habits and do as the nurse anesthetists were accustomed to. Sometimes routines at different workplaces were perceived as hindrances to giving the patient the available heat conservation measures.

*It’s pity that I cannot start the forced-air warming before the operating theatre nurse has scrubbed and draped, at this workplace (18).*

It was also easier to take over a colleague’s habit, instead of reflecting over and changing the routines.

**Table 3:** Overview of dominant (++) and non-dominant (+) ways of understanding heat conservation measures according to Larsson and Holmström’s final steps of the phenomenographic seven-step model [27]

Interview	Preventive	Useable	Untenable	Caring	Adaptive	Routine care
1	++	++	++	+		
2	++	++	++	+		
3	++	++		+		
4	++	++	++	+		
5	+	+	+	++	++	++
6	++	+	+		++	++
7	+					+
8	++	++	++		+	+
9	++	+		+	++	
10	++	++	+	+		
11	+	++	++	+	+	+
12	++	+	+		++	
13	+	++	++	+	++	
14	++	++	++	++	++	+
15	++	++	+		++	
16	+	++		+	+	+
17	++	+			+	+
18	+	++	+	+	++	++
19	++	+	+	+		

## Discussion

The results show that nurse anesthetists have six different ways of understanding the phenomenon heat conservation measures in connection with surgery. The preventive approach was present within all nurse anesthetists, interconnected with one or more approaches. This relationship between these different approaches is flexible depending on the situation. The nurse anesthetists seem to have a mutual goal of protecting the patient from heat loss and maintaining normothermia. In addition, they also aim to help the patient to feel comfortable and cared for. The two approaches, preventive and caring, are closely interconnected, but they are nevertheless distinct. However, the useable, untenable, adoptive, and routine care approaches have an impact on the ability to reach the goals in the preventive and caring approach.

Given the interconnection of the preventive and caring approaches, they are best discussed together. In this study’s result, there is a willingness and a responsibility to give the best care. The goal is to protect the patient from complications and discomfort regardless of which surgery is performed, and this is an expression of the nurse anesthetist’s sense of responsibility for another person, that is, the patient. According to Eriksson the ethics - *arete* is when professionals use wisdom to take responsibility for others and pursue what is good for others [29]. This is also in line with both IFNAʼs codes [18] and the Swedish Patient Safety Act, a law intended to identify potential risks to patients’ safety and thereby protect them from harm and avoid adverse events [30]. Furthermore, *arete* also requires a wholehearted responsibility for the worthy care for the other person [29]. In this study’s results, the nurse anesthetists perceived a feeling of failure when they could not achieve their goal of protecting the patient with heat conservation measures. In addition, when nurse anesthetists and operating theatre nurses couldn’t keep the patient normothermic, despite their best intentions. They experienced silent suffering due to their responsibility in the caring nurse-patient relationship [31].

Furthermore, in the preventive approach, the nurse anesthetists perceived a need to collect data for support before a decision, but mostly as external data. Moreover, in the caring approach, the awakened patients’ vocalized temperature comfort was important. According to Nilsson and Jaensson anesthetic nursing’s *keep in touch* requires listening to the patient’s story and desires, needs, and feelings during the whole perioperative process [21]. To promote the individual patients’ health and well-being, and provide patient-centered care [18]. In this study’s results, this responsibility is perceived mostly on a physiological level during general anesthesia, when they no longer can reach the patients’ needs for temperature comfort and well-being, which continues until they are fully conscious. There is a need for nurse anesthetists to estimate the patients’ needs for protection and warmth. According to Meyer and Lavin, the nurse must identify and pay attention to clinical observations and signals, i.e., vigilance, *the essence of caring in nursing.* There is also a need to calculate risks, be ready, and act efficiently to minimize risks and potential threats [32]. This is referred to as *watch over and be one step ahead* in anesthetic nursing and includes the nurse anesthetist doing what the patients cannot do for themselves [21]. In this study’s results, the nurse anesthetists also use their clinical gaze to observe and collect further signals beside the vital parameters, to act on the total calculated risk to minimize threats to the patient’s health and well-being. The willingness show an intention and motivation to fulfill the goal of protecting the patient. Therefore, it is important to maintain and encourage the willingness of nurse anesthetists, which can be influential in the future implementation of evidence-based heat conservation measures.

In this study’s results, in the useable approach, the nurse anesthetists perceived uncertainty around using heat conservation measures due to their effectiveness, as well as a fear of harming the patient. Contributory factors seem to be a lack of available education and awareness of heat conservation measures, both at a university level and organizational level. That leads to doubts concerning using some of the heat conservation measures. However, guidelines from Germany/Austria, and the UK have made financial calculations, that recommendations are cost-effective in comparison to the cost of perioperative complications [8, 9]. A similar calculation has been done in Australia that shows great cost savings [33]. According to the Swedish Health Care Act, care given should promote health and patient safety [34]. Therefore, it is of important that educators in nurse anesthetist programs offer education about perioperative hypothermia and evidence-based heat conservation measures. Furthermore, at an organizational level, offer lifelong education, evidence-based updates, and guidelines to help the nurse anesthetists provide care. This support to nurse anesthetists can enable, rather than hinder, the use of heat conservation measures.

In the untenable approach in this study, time is perceived as a factor that can hinder the nurse anesthetists using heat conservation measures. Nurse anesthetists do not have enough time to prepare between short surgeries. Similar results are seen when operating theatre nurses do not have enough time for assessment and performance of skin preparation, due to enhanced productivity, and it creates concern for patient safety [35]. Furthermore, in the untenable approach, the nurse anesthetists perceived financial constraints that made them reflect on costs together with risk. This lack of time and financial constrictions is a potential hindrance to increasing the use of heat conservation measures, which needs to be addressed at the organization level, including leadership and organizational structure. Moreover, in the routine care approach, they perceived a time limit: surgery must be longer than 30–60 min to measure temperature or to provide some of the heat conservation measures. These two are interconnected when the nurse anesthetist makes decisions. Similar findings are seen where anesthesiologists also have a time limit due to the uncertainty regarding their effectiveness in relation to time [22], and the benefits of shorter surgeries [22, 36]. According to UK guidelines, the duration of anesthesia is an important factor in the decision to provide heat conservation measures, not only the surgical time. All patients with more than 30 min of anesthesia time should have active warming, preferably as forced-air warming [9].

In the adoptive approach, nurse anesthetists felt split among patients, team members, and the manufacturers information due to the mutual reliance on different professionals. To solve this, they adapted to the situation to avoid friction while retaining their goal to protect the patient, and thereby compensate as well as possible. Similar results were found among anesthesiologists, where some other team members could be experienced as a source of conflict or a hindrance to providing good temperature management [22]. There is complexity when several professions with different specialties work together on a team and are dependent on each other. The team provides care at the same time, but each member focuses on different aspects of the patient’s need for care [37]. To create safer care, there is a need to briefly share information and every team member’s plans for the patient, to create an opportunity to discuss and solve problems before the process starts [38]. To facilitate and improve for future patients, the team can use standardized strategies together with communication about the patient outcomes and give feedback on their own and each other’s performance afterward [22]. Therefore, it is important that all team members have the same awareness about heat conservation measures, and have a common goal and plan.

In this study’s results, in routine care approach, it is perceived that decisions sometimes are taken for granted, based on own habits and internal cultural traditions. Certain types of surgery, of certain lengths, and patients with certain risk factors are interpreted to require more active heat conservation measures. Similar findings show that it was no routine to take the temperature for all surgeries; this depended on the type of surgery, the duration, and the patient’s risk factors [31]. Furthermore, in Boet et al., it also depends on the length of surgery and available equipment [22]. According to Honkavuo and Loe, it is a subjective clinical assessment that is behind the action to provide heat conservation measures. This depends on the nurses’ experience, established rules and routines, and the perioperative units’ internal culture and norms. A routine can be established consciously or unconsciously when repetitive actions are not reflected [31]. Routine care can hinder the use of heat conservation measures from a person-centered perspective.

### Strengths and limitations

In a qualitative analysis, trustworthiness is reflected in terms of five criteria: credibility, transferability, dependability, confirmability, and reflexivity [39]. Credibility: A phenomenographic approach is credible to reach the nurse anesthetists’ perceptions of the phenomena in this study. The choice of excluding nurse anesthetists with less experience than one year is strengthened by Benners’ theory according to which these nurses are seen as advanced beginners and without having the opportunity to capture the whole situation [40]. The interview guide was open-ended and semi-structured to guarantee the same questions to all participants: Follow-up questions were asked to reach more variations of the phenomenon, which together strengthened credibility. One limitation in the interview guide was the words passive and active warming, words frequently used in this research context. These terms were changed to heat conservation measures, this clarification for the nurse anesthetists probably did not affect the credibility in a negative sense. All interviews were held by the first author, and they were reflected on and evaluated by the last-listed author, to strengthen credibility. Data was collected at different times of day, different months of the year, and from participants from four different hospitals, thus increasing the triangulation of data collection and therefore the credibility. The research team had mutual professional competence and a high level of methodological expertise in phenomenography. During the analysis process, there were discussions and reflections at every step, which together increased the credibility. Transferability: The rich variation in age, gender, and work experience gave the findings sufficient transferability to similar contexts, and to anesthesiologists in countries that work with an anesthesia nurse, due to the similar responsibilities as a nurse anesthetist, in relation to heat conservation measures. Dependability: 20 participants are usually sufficient to identify different perceptions [27]. However, a total of 19 nurse anesthetists participated and after 15 interviews no new description was revealed. The rich variation in age, gender, and work experience strengthens the trustworthiness of the results. The dominant and non-dominant ways of understanding the phenomenon were sought for and identified in all participants. Confirmability: A clear description of every step in the process and consensus in the research team was achieved. The categories are presented with extracted quotations to show relevance, which strengthens trustworthiness. Reflexivity: There was no relationship between the participants and the authors. The process included both individual reflections by the first author and reflections together with the research team.

## Conclusion

The results show new knowledge of nurse anesthetists’ perceptions, that may enable or hinder them from using heat conservation measures. Nurse anesthetists intend to prevent patients’ heat loss and maintain normothermia regardless of the type of surgery. This willingness, motivation, and intention are enablers in planning and using heat conservation measures. However, there are perceptions that have an impact on use. Lack of knowledge and guidelines is expressed as doubts and uncertainty and/or routines or habits that can hinder the use of heat conservation measures. When planning for patients’ thermal comfort, nurse anesthetists are recommended to calculate anesthesia time and not only surgical time. Furthermore, use forced-air warming, which is both the most effective and cost-effective and preferably before anesthesia. At an organizational level that includes leadership and organizational structure, there is a need for support to minimize hindrances such as time and financial constraints, access, and not having the right preconditions. To increase knowledge, educators at universities must offer education about perioperative hypothermia and evidence-based heat conservation measures. At the local and national organizational levels, it is necessary to promote lifelong education, updates, and guidelines. This is the first study about nurse anesthetists’ perceptions of heat conservation measures; further research on this is needed as well as research about team members’ perspectives.

## Data Availability

The datasets used and/or analyzed during the current study are available from the corresponding author upon reasonable request.
